# Dysregulation of Lipid Metabolism in Aging Meibomian Glands and Its Molecular Markers

**DOI:** 10.3390/ijms241713512

**Published:** 2023-08-31

**Authors:** Igor A. Butovich, Amber Wilkerson, Seher Yuksel

**Affiliations:** 1 Department of Ophthalmology, University of Texas Southwestern Medical Center, Dallas, TX 75390-9057, USA; amber.wilkerson@utsouthwestern.edu (A.W.); seher.yuksel@utsouthwestern.edu (S.Y.); 2 Graduate School of Biomedical Sciences, University of Texas Southwestern Medical Center, Dallas, TX 75390-9057, USA

**Keywords:** aging, Meibomian gland, meibum, meibogenesis, metabolism, lipid homeostasis, lipid markers, liquid chromatography–mass spectrometry, metabolism, eye diseases

## Abstract

The main function of exocrine Meibomian glands (MGs) is to produce a lipid-rich secretion called meibum which plays a critical role in maintaining the ocular surface homeostasis of humans and most mammals. The chemical composition of meibum, and its quantity produced by MGs, largely determine whether it can fulfill its role successfully. Aging was frequently associated with the onset of various MG-related pathologies. The goal of this study was to determine how aging affects the chemical composition and quantity of meibum in mice, and identify possible molecular markers of aging. Unbiased, untargeted and targeted lipidomic evaluation of mouse MG lipids was conducted using liquid chromatography—high-resolution mass spectrometry, and the results were analyzed using Principal Component, Orthogonal Projections to Latent Structures Discriminant, and Partial Least Square Discriminant Analyses. We found that aging leads to dysregulation of lipid metabolism in MGs, changing the ratios of major classes of MG lipids (such as wax esters, triacylglycerols, and phospholipids) in a progressive manner. Several lipid species that belong to these groups of MG lipids are proposed as clear markers of aging in a mouse model.

## 1. Introduction

Exocrine Meibomian glands (MGs) are embedded in the tarsal plates (TPs) of the upper and lower eyelids of humans [1] and most terrestrial mammals. MGs produce a lipid secretion (also known as meibum [2]) that is delivered onto the ocular surface through a system of ducts and orifices during the blink and/or spontaneously due to the elevated intraductal pressure that is created by newly, and constantly, produced meibum in the ascini of MGs. Meibum, and its main component Meibomian lipids (MLs), play a critical role in maintaining the ocular surface homeostasis [3,4,5,6]. In healthy human subjects and wild type mice, the chemical composition of meibum has been shown to be rather stable [7,8,9,10,11]. However, MLs changed dramatically in the developing MGs of mice from postnatal stages P0 to P30, as did their TP transcriptomes [12]. Moreover, dysregulated lipid metabolism in the MGs of human subjects and mice was demonstrated to be either linked to, or be responsible for, various ocular pathologies [13,14,15,16]. As the chemical composition of meibum, and its quantity produced by MGs, largely determine whether it can fulfill its protective role successfully, monitoring these parameters can provide information on the onset, type, and development of the pathologies. Importantly, many epidemiological studies have demonstrated that aging is commonly associated with the onset of various MG-related pathologies [17,18,19,20,21,22]. However, the exact molecular mechanisms of these pathologies remain, for the most part, either unknown or controversial, and even their diagnosis is a challenging task due to the lack of established molecular markers of the conditions. While the MG lipidome has been shown to undergo remarkable, well-characterized changes during the development of the glands in wild type mice from P0-P3 to about P30 [12], the effects of aging on older mice have not been studied to the same extent, and the findings have been based mostly on physiological, histological, and histochemical observations [23,24,25,26,27], or experiments not designed to characterize MG lipidomes [24,28,29,30,31]. 

Various effects of aging on human meibum have been described in previous publications, e.g., [16,32,33,34,35,36,37] and others, a full analysis of which goes beyond the scope of this paper. However, no comprehensive lipidomic profiling of meibum collected from aging, but otherwise healthy, human subjects has been performed. In a recent paper [7], we explored this matter using liquid chromatography–high-resolution quaternary time-of-flight mass spectrometry (LC–MS). Our findings demonstrated only minimal differences between the study groups. Specifically, only incremental changes in some of the lipid classes (such as cholesteryl esters of *(O)*-acylated ω-hydroxy fatty acids (Chl-OAHFA)) and α,ω-diacylated diols were observed, and could mostly be characterized as “trends”. However, the two groups that were evaluated in that study were subjects of 29 ± 5 years and 68 ± 7 years of age, which did not cover the oldest groups of adults, and did not allow us to visualize the possible intra-group age-related changes in their MG lipidomes. In a follow-up publication [16], statistically significant differences in some polar and nonpolar lipids were reported for, seemingly, the same two age groups (25.7 ± 3.8 and 58.4 ± 7.5 years old); thus, the vector of changes was not established either. Therefore, the main goal of this study was to determine how aging affects the chemical composition and quantity of meibum in mice, and identify potential, easily detectable molecular markers of aging in wild type mice using lipidomic approaches.

## 2. Results

### 2.1. Unbiased, Untargeted Analysis of Mouse Meibomian Lipids

Mouse ML samples from P17 to P730 mice were collected (Table 1) and analyzed as described earlier [12]. Since no single LC–MS procedure can cover the whole range of MLs due to their extreme diversity, several different LC–MS procedures were used to detect as many lipid classes as possible: a C_18_ reverse phase gradient LC with electrospray ionization (ESI) MS detection in positive and negative ion modes (PIM and NIM, respectively), a C_18_ reverse phase gradient LC with atmospheric pressure chemical ionization (APCI) detection in PIM, and a C_8_ reverse phase isocratic LC with detection using APCI PIM and ESI NIM. Note that ESI experiments are better suited for analyzing more polar lipids, for example free fatty acids (free FA), phospholipids (PLs), sphingomyelins (SMs), and similar compounds, while the APCI approach is more sensitive toward less polar and neutral lipids such as wax esters (WEs), squalene, and free cholesterol (Chl).

**Figure 1:**
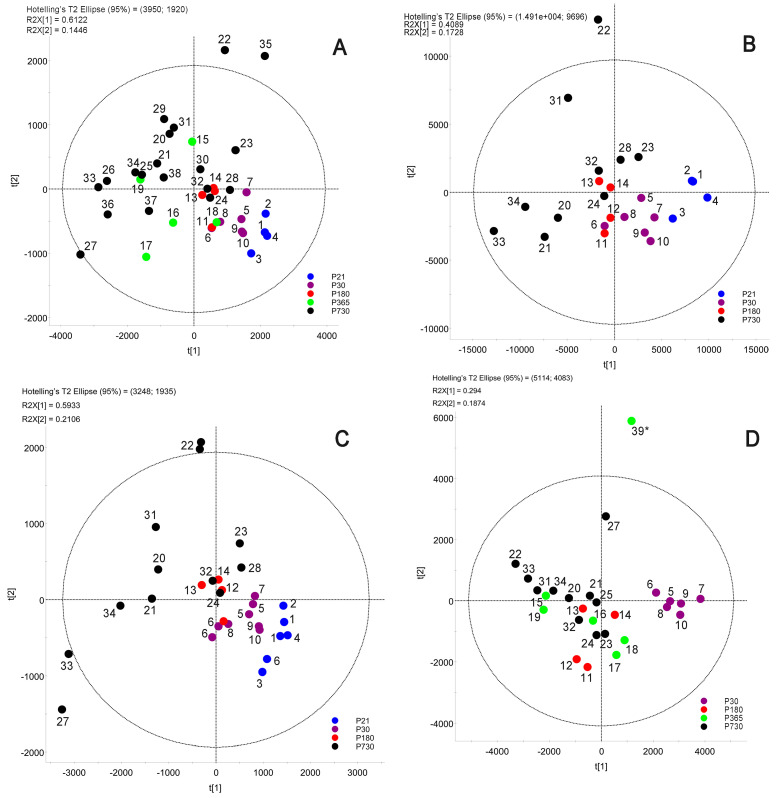
PCA analysis of the Meibomian lipidomes of aging mice. Samples were analyzed using gradient C_18_-LC—MS APCI PIM (**A**), gradient C_18_-LC—MS ESI PIM (**B**), isocratic C_8_-LC—MS APCI PIM (**C**), and gradient C_18_-LC—MS ESI NIM (**D**). Blue dots, P21 samples; purple dots, P30 samples; red dots, P180 samples; green dots, P365; black dots, P730 samples. Additional information on the samples is included in Supplemental Table S1. Samples 1, 5, 6, and 22 in Panel C are shown in duplicates to demonstrate reproducibility of the analyses. See References [38,39] for explanations on the use of PCA for metabolomics data analysis. *—a strong outlier.

Initially, the samples were analyzed using C_18_-LC—MS APCI PIM and processed using a Progenesis QI software package from Nonlinear Dynamics/Waters Corp. (v.2.3, Milford, MA, USA). The results were exported into EZinfo (v.3.0.3; Waters Corp.), processed initially using its unbiased Principal Component Analysis (PCA) routine (a commonly used variant of a multivariate statistical analysis protocol [38]), and plotted as a Scores plot (Figure 1A). The analysis of the data demonstrated apparent changes in the MG lipidomes of aging mice. While there was a clear but moderate shift in the MG lipidomes from P21 (young adults) to P180 (matured adults), which formed tight clusters with minimal intergroup separation, past P180 the age-related differences became more prominent, and the spread widened: a clear sign of a higher variability in the MG lipid composition in the animals of the advanced age groups. Importantly, the P365 and P730 samples from aged mice showed the widest spread of parameters with some of the specimens overlapping with the P30 and P180 groups. The highest variability was observed for the oldest P730 group of mice. Notably, P70 specimens from a previous study (10 males and 10 females) showed tight grouping with no measurable effect of sex on meibogenesis [9].

Notably, similar results were obtained in the C_18_-LC—MS ESI PIM and C_8_-LC—MS APCI PIM experiments (Figure 1B,C). Then, the samples were analyzed in the ESI NIM to check for the presence of anionic Meibomian lipids (Figure 1D), and the grouping of the samples was found to be close to that determined in the experiments in PIM.

**Figure 2:**
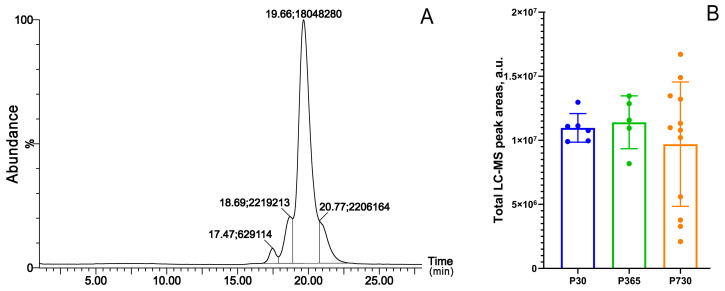
Comparison of the total lipid pool in young (P30), mature (P365), and old (P730) mice using isocratic C_8_-LC—MS APCI PIM. (**A**) A representative total ion chromatogram of a P30 sample. The LC—MS peaks are labeled as (Retention time, min; peak area, total ion current; extract from 2 tarsal plates). (**B**) The total LC—MS peak areas are proportional to the total amount of lipids in the sample. Note tight grouping of the samples from young (P30, blue dots/bars) and mature (P365, green dots/bars) mice, and a much wider spread of data obtained with older P730 mice (orange dots/bars). A combined PLS-DA plot for P21—P730 samples is shown in Supplemental Figure S1.

The overall amount of extracted TP lipids per one TP was estimated using their total ion chromatograms (TIC) as described before [7]. The mean values for P30, P365, and P730 mice did not differ (Figure 2), but the spread of the P730 data was significantly wider, showing considerable fluctuations in the MG’s capacity to produce meibum. The data shown in Figure 2B for P30 and P730 passed both the Kolmogorov–Smirnov and Shapiro–Wilk Normality tests, which indicated that the data matched the pattern expected if the data were drawn from a population with a normal distribution. Thus, no evidence of any intra-group clustering was found.

**Figure 3:**
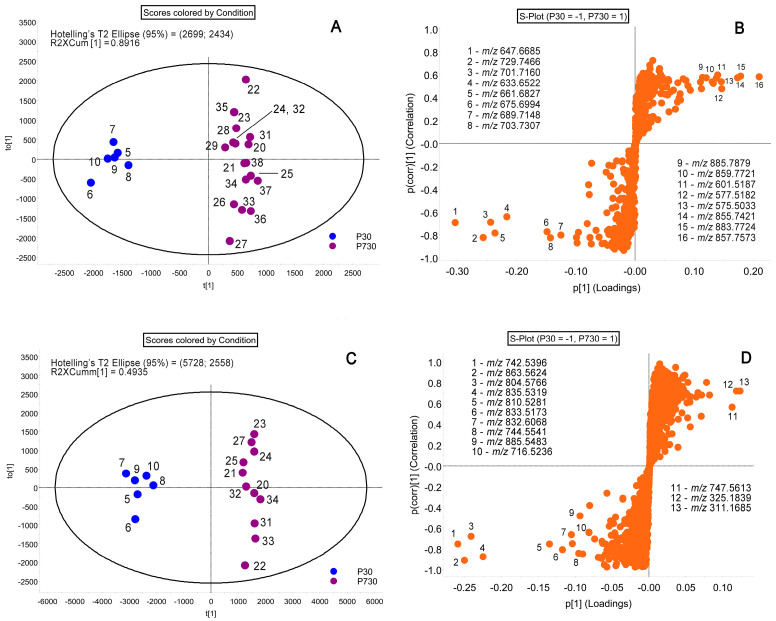
OPLS-DA analysis of mouse Meibomian lipidome of young and aging mice. Samples were analyzed using gradient C_18_-LC—MS APCI PIM (**A**,**B**) and C_18_-LC—MS ESI NIM (**C**,**D**). Two age groups, P30 and P730, were compared and produced two separate groups with high intergroup differences in the Scores plots (**A**,**C**). Note tight grouping of samples from younger P30 mice, and a much wider spread of data obtained with older P730 mice. From S-plots for P30 (**B**) and P730 (**D**) groups of samples, analytes with the highest impact on the separation of the study groups, i.e., potential biomarkers, were identified.

As the overall number of detected LC–MS signals (i.e., potential lipid analytes whose abundances exceeded 0.01% of the intensity of the base peak in the sample spectrum) approached 1000 or more, manual analysis of these massive data as an initial step of the project was considered impractical, and a different approach, Orthogonal Projections to Latent Structures Discriminant Analysis (OPLS-DA; a part of Progenesis QI) of the raw LC–MS data, was chosen instead. This approach is known to allow for identification of the analytes that have the largest impact on the separation of the study groups. As only two groups can be compared at the same time using OPLS-DA, the P30 and P730 specimens were chosen as those that show clear differences between the age groups (Figure 3). The Scores plots of the samples recapitulated the data shown in Figure 1: specifically, tight grouping of the P30 samples, a much wider spread of the P730 samples, and a clear intergroup separation of the P30 and P730 specimens along the *X*-axis.

The most significant variables (i.e., lipid analytes) with the highest impact on the separation between the P30 and P730 groups were identified from the S-plots (Figure 3B,D). Per Wiklund et al., the S-plot “...visualizes both the covariance and correlation between the metabolites and the modeled class designation. Thereby the S-plot helps identifying statistically significant and potentially biochemically significant metabolites, based both on contributions to the model and their reliability.” [39]. This approach is a standard protocol implemented in the Progenesis QI software package and, therefore, was used in our study to identify potential biomarkers of aging among Meibomian lipids. The most reliable potential biomarkers for each of the age groups would be those with a high magnitude (i.e., the most distant from the origin of the graph on the p [1] axes, and the most distant from the origin on the p(corr) [1] axes), and positioned in the lower left (for P30) and the upper right (for P730) quadrants. 

**Figure 4:**
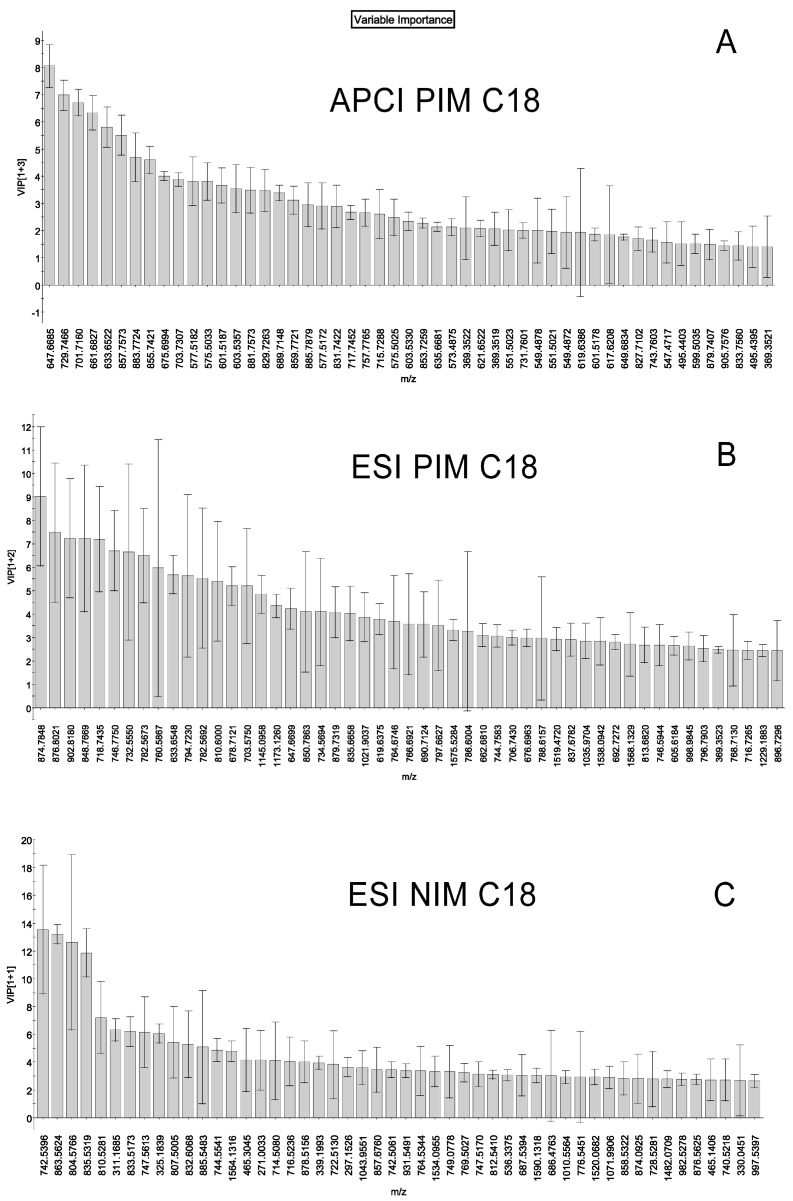
The Variable Importance Plots (VIPs) of the samples shown in Figure 3. The plots were generated in the EZInfo. Results of experiments in three C_18_-LC—MS modes are shown: (**A**) APCI PIM, (**B**) ESI PIM, and (**C**) ESI NIM. The analytes with the highest impact on the separation of the P30 and P730 study groups and the lowest standard errors can be identified from the charts. The chemical nature of the major compounds—markers of the conditions—was evaluated using their LC retention times, fragmentation patterns (using the MS^E^ functionality of the Waters’ MS^E^ Data Viewer v. 1.4), and their elemental composition (using EleComp routine of the Progenesis QI software package). VIP(1+1), VIP(1+2), and VIP(1+3) are VIP scores (unitless parameters) [40]. General information on VIPs can be found at https://www.sartorius.com/download/544940/simca-15-user-guide-en-b-00076-sartorius-data.pdf (accessed on 25 July 2023). Additional information on the chemical nature of Meibomian lipids can be found in our previous publications [9,10]. Note that not all of the signals are those of lipids as some of them may have originated from nonlipid compounds.

**Figure 5:**
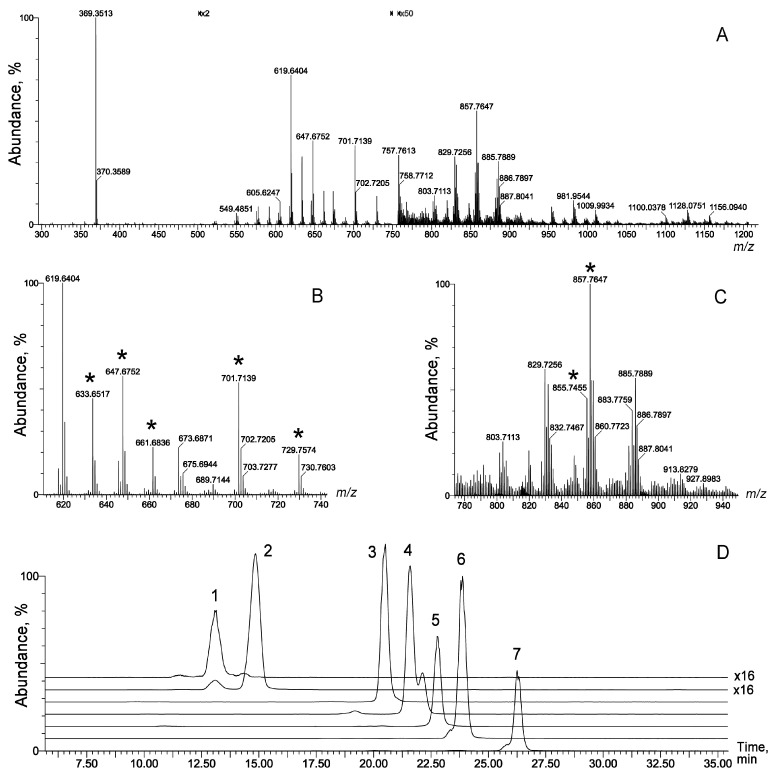
(**A**–**C**) APCI PIM mass spectra of a representative P30 sample; (**D**) extracted ion chromatograms (EIC) of seven marker lipids shown in Table 2: WE C43:1 (1), WE C44:1 (2), WE C45:1 (3), WE C48:2 (4), WE C50:2 (5), TAG 52:4 (6), and TAG 52:3 (7). Selected markers are labeled in (**B**) and (**C**) with asterisks (*). Note that some portions of the graphs (**A**–**C**) were magnified ×2 (*m*/*z* 500 to 750) and ×50 (*m*/*z* above 750) times, to compensate for the lower abundance of signals in those areas, as were EIC of markers 1 and 2 in (**D**).

To verify these findings, visualize the analytes with the lowest errors, and eliminate less influential analytes and those with high errors from further analyses, Variable Importance Plots (VIPs; reviewed and explained in [40]) were used (Figure 4). A list of the most influential compounds found in our experiments is shown in Table 2. Representative APCI PIM mass spectra of a mouse sample with seven labeled markers and their corresponding extracted ion chromatograms (EIC) are shown in Figure 5. Therefore, these markers were selected for more targeted LC–MS analyses of the study samples.

### 2.2. Targeted Analysis of Mouse Meibomian Lipids

**Figure 6:**
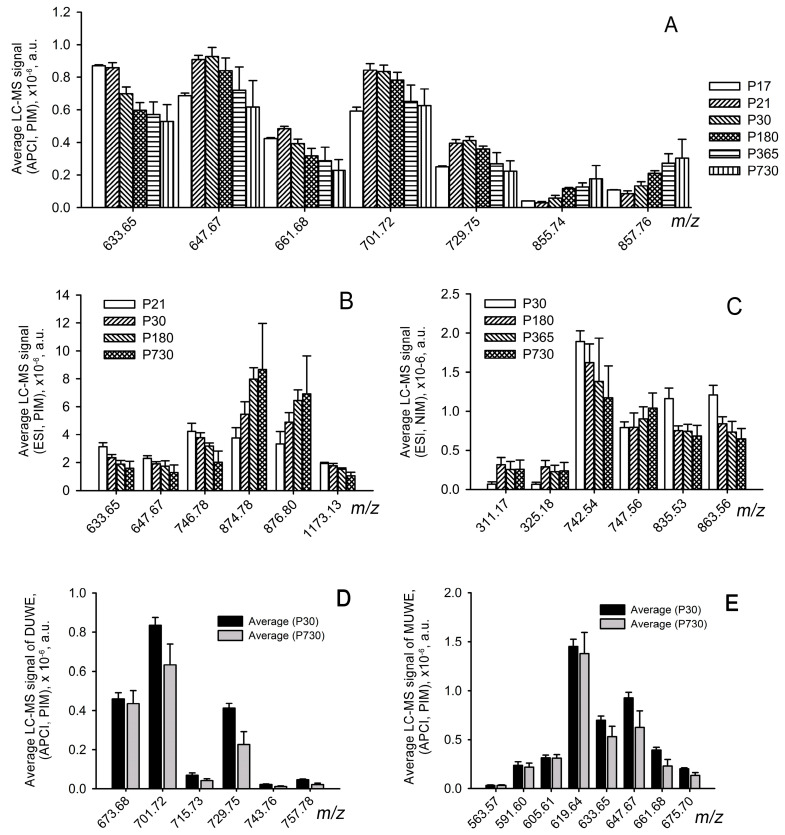
Targeted LC–MS analysis of marker tarsal plate lipids in P17 to P730 mice. (**A**) C_18_-LC—MS APCI PIM; (**B**) C_18_-LC—MS ESI PIM; (**C**) C_18_-LC—MS ESI NIM. The analytes for each condition are listed in Table 2 and Supplemental Table S2. Changes in the levels of accumulation of diunsaturated wax esters (DUWE) and monounsaturated wax esters (MUWE) for P30 and P730 sample groups are shown in (**D**,**E**). Data are presented in the (mean ± standard deviation format). Study animals/samples are listed in Table 1 and Supplemental Tables S1 and S2.

The dynamics of the changes in the potential marker lipids from Table 2 and Figure 3, Figure 4 and Figure 5 with aging are summarized in Figure 6A–C. First, the APCI PIM data were analyzed for all the tested age groups from P17 to P730. The signals of extremely long-chain C_43_–C_50_ WEs showed a general tendency to rise from P17 to P30, which corroborated our earlier results [12], but became progressively lower with aging after P30. At the same time, regular C_52:3_ and C_52:4_ TAGs that are typical of human and mouse blood plasma steadily increased from P17 to P730. The same changes in WEs and TAGs were observed in ESI PIM experiments, though the instrument responses were not identical to those in the APCI PIM due to the differences in the ionization techniques. Finally, the ESI NIM experiments allowed the status of more polar lipids, such as PLs, ceramides, and others, to be monitored. A few very polar compounds, e.g., analytes with *m*/*z* values of 297.16 ± 0.01, 311.17 ± 0.01, 325.18 ± 0.01, and 339.19 ± 0.01 that had been detected in the S-plots and VIP’s of all study samples, were identified, in fact, as linear alkylbenzenesulfonates. The latter compounds are typical contaminants of the surfactant origin that are found in the environment as pollutants and can possibly accumulate in human and animal bodies via direct exposure or through the consumption of water and food [41,42]. Consequently, these analytes were excluded from further consideration. The other selected compounds were positively identified as PLs: a phosphatidylethanolamine PE36:2 and two phosphatidylinositols, PI34:1 and PI36:1. The compound with *m*/*z* 747.5613 was classified as C45:8 (FA residues only) TAG. As with other TAGs listed in Table 2, this analyte was upregulated in older mice.

An important trend was observed while analyzing elongation patterns of monounsaturated and diunsaturated WEs (MUWEs and DUWEs, correspondingly): the analysis demonstrated a steady and statistically significant downregulation of longer-chain MUWEs with *m*/*z* values at, or above, 619 (i.e., ≥C_42_H_82_O_2_) and DUWEs with *m*/*z* values of above 673 (>C_46_H_88_O_2_) in aged mice (Figure 6D,E).

Thus, the markers described above seem to follow the same patterns regardless of the method of the analysis: (1) the levels of specific WEs, PEs, and PIs are increased in younger mice, while TAGs are increased in older mice, and (2) the biosynthesis of extremely long-chain MUWEs with carbon chain lengths ≥ C_41:1_ and DUWEs longer than C_46:2_ is downregulated in older animals.

### 2.3. The Ratios of Lipid Markers

The age-related changes described above were noticeable and, in many cases, statistically significant. Possible markers of aging derived from the S-plots and VIPs were evaluated, with P730/P30 ratios shown in Table 3 to illustrate the trend. Three different LC–MS procedures were tested. Other combinations of markers can be selected from the S-plots and VIP graphs, but those included in Table 3, in our hands, combined rather high abundances and low errors (Figure 3, Figure 4, and Figure 6).

**Figure 7:**
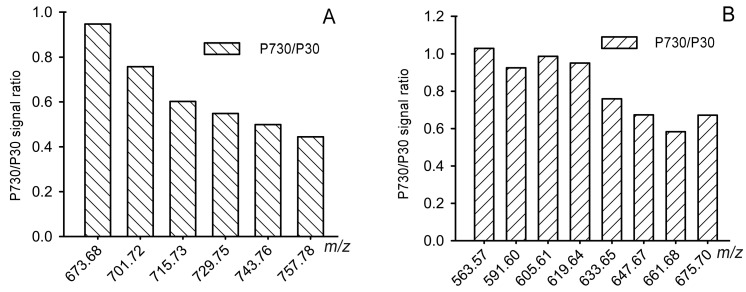
The P730/P30 signal ratios of selected marker lipids illustrate a tendency of extremely long-chain diunsaturated (**A**) and monounsaturated (**B**) wax esters to decline with aging. Study animals/samples are listed in Table 1. Sample IDs and their LC–MS signals are shown in Supplementary Tables S1 and S3.

This approach was used to study the effects of aging on the elongation patterns of Meibomian WEs (Figure 7). The P730/P30 ratios vs. *m*/*z* plots revealed an apparent correlation between the P730/P30 ratios and the lengths of the WEs, with the largest two-fold decline in the longest C_52:2_ DUWE, and almost no impact on the shortest WE C_46:2_ shown in Figure 7. In case of MUWEs, the change did not start before C_43:1_ and continued with elongation of the compounds to, at least, C_46:1_. These observations clearly connected the activity of the FA elongation cycle in the MGs of mice with their aging.

However, the use of the chosen parameter, the ratios of signals of a specific lipid in ML samples from two age groups, is dependent on several parameters that can have an impact on the results of the experiments. Parameters that are easy to control include the number of mouse TPs per lipid sample (N), the final volume of the sample stock solution (in mL), and the LC–MS injection volume (in μL), which can either be kept constant throughout the experiment, or the results can be normalized, for example, per one TP, one mL of the sample solution, and one μL of the injected sample. However, this approach cannot account for the differences in the physical size of the collected specimens (the TPs are small and quickly desiccate losing their weight), and other inadvertent changes in the experimental conditions. The use of external calibration curves or internal standards is hampered by the sheer diversity of MLs and the current lack of proper standards of complex lipids with extremely long-chain saturated and unsaturated FA and fatty alcohol (FAl) residues, which the meibum of humans and mice is known for [2,11,43,44].

**Figure 8:**
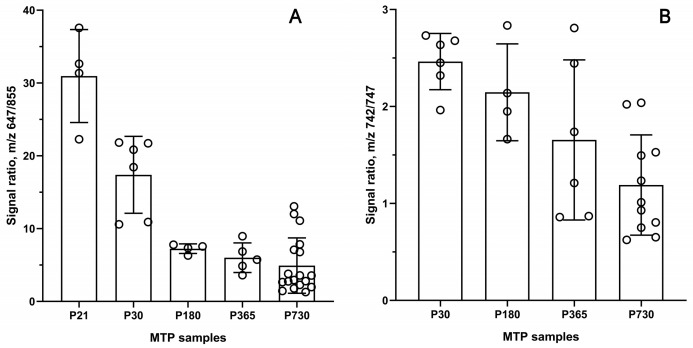
Signal ratios of marker lipid pairs 647/855 (**A**) and 742/747 (**B**), which displayed opposite trends in aging Meibomian glands. Data are presented in the mean ± standard deviation format. LC–MS data can be found in Supplementary Table S4.

In order to compensate for some of these hindrances, we decided to calculate the *ratios* of two (or more) markers within each sample measured in the same LC–MS runs. Moreover, choosing the markers with *opposing* trends (up and downregulated) to calculate their apparent ratios could, theoretically, help with finding and visualizing the trends in TP lipid metabolism changes that are associated with aging (see Section 3. Discussion). This approach resulted in a remarkable differentiation between younger and older mouse TPs (Figure 8A), approaching, on average, a six-fold difference in the signal ratios of the C_44_H_86_O_2_ WE and C_55_H_98_O_6_ TAGs for P21 and P730 specimens. 

Similar results, though with smaller differences, were obtained for other markers, for example, a PE and a TAG (Figure 8B). We believe that many other analytes whose combinations could be optimized on the bases of their overall abundances, low experimental errors, largest differences between their abundances in the study groups, and biological relevance can be used for the same purpose in many other types of samples. However, measuring the WE/TAG ratios for mouse Meibomian lipids revealed that aging has an impact on the efficacy of meibogenesis [11]; specifically, on the biosynthesis of extremely long-chain (ELC) WEs and accumulation of TAGs in the mouse TP tissues.

### 2.4. mRNA Expression Levels of Major Genes of Meibogenesis

As the efficacy of lipid metabolism in developing and maturing MGs strongly depends on the level of expression of the major genes of meibogenesis [12], similar comparisons were made for young (1–3 mo old) and aged (32 mo old) mice. Importantly, of all tested genes, only a handful of them, specifically, *Elovl1*, *Elovl3, Elovl6, Elovl7*, and *Fasn*, were shown to be expressed differently with a statistical significance of *p* < 0.05 (Table 4). In fact, *Elovl1*, *Elovl3,* and *Elovl7*, which encode elongases of very, and extremely, long-chain FAs with carbon chains longer than C_18_-C_20_, were downregulated, while *Elovl6* and *Fasn* that synthesize shorter-chain FAs were somewhat upregulated. The genes that encode the formation of more complex lipids via esterification reactions, such as *Awat1/Awat2, Soat1/Soat2,* and *Dgat2*, as well as those that are involved in the biosynthesis of Chl (*Dhcr7/Dhcr24*), FA desaturation (*Scd1-Scd4*), and formation of FAls (*Far1/Far2*) were not affected to a measurable extent.

## 3. Discussion

One of the main decisions to make when conducting comprehensive characterization of complex mixtures of analytes of biological origin is the choice of the analytical technique. The use of LC–MS compared to other techniques, such as infrared (IR), Raman, or nuclear magnetic resonance (NMR) spectrometries [45,46,47,48,49,50] offers, among other advantages, a clearer view of the changes in the individual homologous lipids, such as series of WEs, TAGs, cholesteryl esters, etc., with different elongation and unsaturation patterns. Indeed, differentiation between individual WEs in complex mixtures without resorting to LC–MS or gas chromatography–mass spectrometry (GC–MS) is an all but impossible task unless a prior separation of lipids has been performed, in which case the use of LC or GC in the first place becomes a more practical choice. Moreover, quantitation of individual lipids in mixtures of homologous compounds (which almost all Meibomian lipids are) is virtually impossible due to the overlap in their characteristic NMR, IR, or Raman signals. Any direct infusion MS approaches are also limited in their ability to differentiate between isobaric compounds that belong to the same or different lipid groups because of the overlap in their MS signals and a loss of a critically important identifier—a GC or a LC retention time. Thus, to elucidate the changes in complex lipid metabolism pathways (such as meibogenesis) and find molecular markers of specific conditions, the LC–MS approach seems to be an indispensable tool with the highest selectivity and sensitivity available at this time. 

Notably, the use of IR, Raman, and NMR spectroscopies for meibum studies flourished only after the detailed characterization of Meibomian lipids had been conducted using either classical experimental techniques [2,51,52,53,54], or LC–MS and GC–MS analyses of intact lipids [55,56]. Moreover, the sensitivity of modern LC–MS techniques is considerably higher than that of other non-MS-based approaches, which allows for the reliable detection and identification of individual lipid species in complex mixtures at very low concentrations unapproachable by other means.

However, the use of other spectrometric techniques does provide advantages over LC–MS and GC–MS in such areas as the biophysical characterization of meibum and similarly complex lipid mixtures, estimation of the overall, lipid-class-independent ratios of straight-chain and branched lipids, the detection of lipids with conjugated double bonds, etc. Consequently, the ultimate choice in choosing the best experimental technique should be based on the questions to be answered. 

In this project, the age-related changes in the Meibomian lipidomes of mice were studied using several LC–MS techniques tailored for specific lipid classes and groups. One of the main outcomes of our experiments was an observation that the Meibomian lipidomes of younger experimental animals formed tight, highly clustered groups on PCA and OPLS-DA plots, while their spread remarkably widened as the mouse aged (Figure 1). The oldest groups of the animals, P730 and P365, produced a wide variety of specimens, some of which overlapped with (or came in close proximity to) P180, or even P30 groups. These results were an indication that in some animals, meibogenesis was almost unaffected by their advanced ages, while in others, it was considerably altered. 

The changes in the specificity of meibogenesis were attributed to: (1) specific WE/TAG and PE/TAG balances, which were lower in aged mice, and (2) abnormal WE elongation patterns, which shifted toward shorter and more saturated WEs as mice aged (Figure 3, Figure 4, Figure 6, Figure 7 and Figure 8). As TAGs are considered to be precursors of typical MLs and serve as sources of carbon and energy for meibogenesis, their upregulation in the TPs of older mice can either be a result of the abnormal accumulation of the TAGs, or a decline in the biosynthesis of ELC WEs. The mean amounts (Figure 2) of MLs that were produced in the TPs of young and old mice were quite similar, but the P30 group showed a much tighter grouping of the samples than the P730 group.

Another important parameter to discuss is the choice of markers of aging. From our standpoint, the most prudent approach is to start with the unbiased analysis of the data using PCA, and then continue the analysis using PLS-DA or OPLS-DA. The first PCA step demonstrates the overall similarities or dissimilarities between the study groups, while the second approach identifies the most influential markers of the conditions. Indeed, some lipids were identified as being specific markers of either young or old subjects (Figure 3B,D). However, in the case of relatively small differences (i.e., small changes in ratios) and/or high variability in the data (i.e., high standard errors), the markers may provide inconclusive results, or be missed altogether. We attempted to overcome this problem by using the signal ratios determined for two, or more, putative markers that display opposite trends in young and aged mice (Figure 8). In fact, the WE/TAG and PE/TAG ratios did show a dramatic, steady, age-dependent decline, and can possibly be used as markers of aging. As a note of caution, the ratios do depend on not only the actual balances of the markers in a given sample but also on the experimental LC–MS techniques, and the conditions of the analyses should be kept unaltered throughout the experiment. Nevertheless, the approach for determining the ratios of two analytes with opposing trends in one and the same experiment should accentuate the trends better than a simple change in just one parameter between two conditions, regardless of the experimental conditions, and can be used in other types of experiments as well.

Our observations of the changes in the ML profiles can be partially explained by the transcriptomic data as aging led to the downregulation of *Elovl1*/ELOVL1, *Elovl3*/ELOVL3, and *Elovl7*/ELOVL7 that are responsible for FA elongation beyond C_16_, and by the upregulation of *Fasn*/FASN and *Elovl6*/ELOVL6, which control the biosynthesis of, correspondingly, saturated and polyunsaturated C_16_–C_20_ FAs, frequently found in TAGs and certain WEs. We hope that these observations will be used and verified in future studies of the effects of aging on ocular physiology in general, and meibogenesis specifically, that are being, or will be, conducted in this and other laboratories.

## 4. Materials and Methods

Lipid standards were obtained from MilliporeSigma (St. Louis, MO, USA), Avanti Polar Lipids Inc. (Birmingham, AL, USA), and Nu-Check Prep, Inc. (Elysian, MN, USA). Organic solvents of HPLC or MS grade were from MilliporeSigma or ThermoFisher (Waltham, MA, USA).

All animal procedures were approved by the UT Southwestern Medical Center Institutional Animal Care and Use Committee and were conducted in accordance with the ARVO Statement for the Use of Animals in Ophthalmic and Vision Research. Experimental animals were wild type C57BL/6 mice. The animals were kept under a 12 hr light/12 hr dark cycle with unlimited access to water and a 2016 Teclad global 16% protein rodent chow (Envigo, Indianapolis, IN, USA). 

To collect Meibomian lipids, between 2 and 4 whole tarsal plates were excised from freshly euthanized animals under a Zeiss Stemi 508 dissecting microscope (Zeiss, White Plains, NY, USA). The tissues were extracted with a chloroform–methanol (2:1, vol/vol) solvent mixture, the extracts were placed in 2 mL glass HPLC autoinjector vials, evaporated to dryness under a stream of nitrogen, redissolved in 0.5 or 1 mL of iso-propanol, and sealed with Teflon-lined caps. The samples were stored in sealed vials at −20 °C or −80 °C before the analyses.

Lipid analyses were performed using reverse phase C_8_ and C_18_ LC–MS as described recently [12]. A Synapt G2-Si high-resolution quadrupole Time-of-Flight mass spectrometer was coupled with an Acquity M-Class ultra-high-pressure liquid chromatograph (all from Waters Corp.; Milford, MA, USA) for isocratic and gradient LC experiments. The Synapt was equipped with either a low-flow ESI, or an IonSabre II APCI ion sources, all interfaced with the mass spectrometer via a ZSpray/LockSpray housing unit. A NitroFlow Lab nitrogen generator was supplied by Parker Hannifin/Parker Balston (Haverhill, MA, USA). All experiments were conducted in “Sensitivity” mode with R ≥ 10,000 FWHM (full width at half maximum). The scan range was 100 to 2000 amu, with a scan rate of 1 scan per second. The typical mass errors were between 1 and 5 mDa. A Leucin/Enkephalin was used as a LockSpray solution to automatically correct the *m*/*z* values. 

Both isocratic and gradient elution experiments were performed. For isocratic experiments, the LC system was equipped with a C8 BEH Acquity column (1.7 μm, 2.1 mm × 100 mm). The gradient experiments were performed on a C18 BEH Acquity column (1.7 μm, 1 mm × 100 mm). For isocratic elution, a solvent mixture of 5% aqueous solution of 10 mM ammonium formate and 95% iso-propanol at a flow rate of 16 μL/min was used. The column temperature was kept at 40 °C. For gradient experiments, a binary gradient of acetonitrile and iso-propanol, both with 5% of aqueous 10 mM ammonium formate [11], also at a flow rate of 16 μL/min, was utilized. The column temperature was set at 35 °C. Less than 1 μL of the sample solutions was injected, to avoid the column and mass spectrometer being overloaded.

The raw data were analyzed in the MassLynx (v.4.1) and Progenesis QI (v.2.3) software packages (from Nonlinear Dynamics/Waters Corp.). The PCA, PLS-DA, and OPLS-DA multivariate statistical analyses were conducted in the Progenesis QI and EZinfo (v.3.0.3) software packages (both from Waters), while targeted analyses of individual lipids were performed in the MassLynx software package (v.4.1, also from Waters) as described before [12]. The Kolmogorov–Smirnov Normality and the Shapiro–Wilk Normality tests were performed, respectively, in SigmaStat (v.3.5) and SigmaPlot (v.11.0) software packages (both from Systat Software, Inc., San Jose, CA, USA).

Mouse TP RNA was collected and analyzed exactly as described in a recent publication [12] using Clariom D mRNA microarrays (from Affymetrix, Santa Clara, CA, USA). The expression data were processed in the Expression Console (v.1.4.1.36) and Transcriptome Analysis Console (v.4.0.1; also from Affymetrix) using the PCA approach.

## 5. Conclusions

We found that aging leads to the dysregulation of lipid metabolism in MGs, changing the ratios of major classes of MG lipids (such as wax esters, triacylglycerols, phospholipids, and some others) in a progressive manner. Several lipid species that belong to these groups of MG lipids are proposed as clear markers of aging in a mouse model.

## Figures and Tables

**Table 1:** Study animals used for lipidomic analyses ^1^.

Mouse Age Group (Days)	Number of Animals, (Males + Females)	Mouse AgeGroup (Days)	Number of Animals, (Males + Females)
P17	2F + 1 sex unknown	P180	4M
P21	3M + 1F	P365 ^2^	1M + 5F
P30	3M + 3F	P730	6M + 13F

^1^ Wild type C57BL/6 mice. ^2^ One female sample from P365 group (#39) was analyzed only in ESI NIM due to high contamination visible in other MS modes. See Supplemental Table S1 for details.

**Table 2:** Suggested Meibomian lipid markers of aging and experimental conditions for their detection.

Protocol	*m*/*z* (exp.)	Lipid Class ^1^	Molecular Formula, Adduct ^2^
C_18_-LC—MS APCI PIM	633.6548 647.6685 661.6827 701.7160 729.7466 855.7421 857.7573	WE C43:1 WE C44:1 WE C45:1 WE C48:2 WE C50:2 TAG 52:4 TAG 52:3	C_43_H_85_O_2_, (M+H)^+^ C_44_H_87_O_2_, (M+H)^+^ C_45_H_89_O_2_, (M+H)^+^ C_48_H_93_O_2_, (M+H)^+^ C_50_H_97_O_2_, (M+H)^+^ C_55_H_99_O_6_, (M+H)^+^ C_55_H_101_O_6_, (M+H)^+^
C_18_-LC—MS ESI PIM	633.6548 647.6699 746.7750 874.7848 876.8021 1173.1260	WE C43:1 WE C44:1 WE C50:1 TAG 52:3 ^3^ TAG 52:2 ^3^ Chl-OAHFA	C_43_H_85_O_2_, (M+H)^+^ C_44_H_87_O_2_, (M+H)^+^ C_50_H_100_NO_2_, (M+NH_4_)^+^ C_55_H_104_NO_6_, (M+NH_4_)^+^ C_55_H_106_NO_6_, (M+NH_4_)^+^ C_79_H_146_NO_4_, (M+NH_4_)^+^
C_18_-LC—MS ESI NIM	742.5396 747.5613 835.5319 863.5624	PE 36:2 TAG C45:8 ^3^ PI 34:1 PI 36:1	C_41_H_78_NO_10_P, (M−H)^−^ C_48_H_77_O_6_, (M−H)^−^ C_43_H_80_O_13_P, (M−H)^−^ C_45_H_84_O_13_P, (M−H)^−^
	311.1685 325.1839	Suspected contaminants: alkylbenzenesulfonates	

^1^ WE, wax ester; TAG, triacylglycerol; Chl-OAHFA, cholesteryl ester of *(O)*-acylated ω-hydroxy fatty acid; PE, phosphatidylethanolamine; PI, phosphatidylinositol. ^2^ The identities of markers were established using their LC retention times, elemental composition, and online databases LipidMaps and Metabolomics Workbench. ^3^ Number of carbons in FA residues only. Additional main lipids are listed in Supplemental Table S2.

**Table 3:** Suggested Meibomian lipid markers of aging and experimental conditions for testing.

Protocol	Molecular Formula	*m*/*z*	Type	P730/P30 Ratios ^1^	*p*-Value
C_18_-LC—MS APCI PIM	C_43_H_84_O_2_ C_44_H_86_O_2_ C_45_H_88_O_2_ C_48_H_92_O_2_ C_50_H_96_O_2_ C_55_H_98_O_6_ C_55_H_100_O_6_	633.6548 647.6685 661.6827 701.7160 729.7466 855.7421 857.7573	WE WE WE WE WE TAG TAG	0.759 ↓ 0.674 ↓ 0.583 ↓ 0.757 ↓ 0.548 ↓ 3.004 ↑ 2.249 ↑	1.04 × 10^−3^ 3.33 × 10^−4^ 1.22 × 10^−5^ 1.79 × 10^−4^ 1.06 × 10^−6^ 3.62 × 10^−3^ 3.46 × 10^−3^
C_18_-LC—MS ESI PIM	C_43_H_84_O_2_ C_44_H_86_O_2_ C_50_H_96_O_2_ C_55_H_100_O_6_ C_55_H_102_O_6_ C_79_H_142_O_4_	633.6548 647.6685 729.7466 855.7421 857.7573 1173.1260	WE WE WE TAG TAG Chl-OAHFA	0.659 ↓ 0.653 ↓ 0.509 ↓ 1.657 ↑ 1.48 ↑ 0.574 ↓	2.40 × 10^−3^ 9.41 × 10^−3^ 1.39 × 10^−4^ 2.37 × 10^−2^ 6.29 × 10^−2^ 1.76 × 10^−5^
C_18_LC—MS ESI NIM	C_41_H_79_NO_10_P C_48_H_78_O_6_ C_43_H_81_O_13_P C_45_H_85_O_13_P	742.5396 747.5613 835.5319 863.5624	PE TAG PI PI	0.594 ↓ 1.251 ↑ 0.566 ↓ 0.509 ↓	7.36 × 10^−4^ 1.24 × 10^−2^ 6.86 × 10^−6^ 6.12 × 10^−7^

^1^ Determined using Descriptive Statistics routine of the Progenesis QI. The numerical values of the LC–MS signals for tested P30 (n = 6) and P730 (n = 18) samples were averaged for each age group, and their P730/P30 ratios were calculated, alongside their corresponding *p*-values. The default parameters of the MassLynx and Progenesis QI (peak response heights), were used to compare the signals. The ratios depend on the analytical procedures and must be estimated using identical experimental conditions. ↑, upregulation; ↓, downregulation.

**Table 4:** Log(2) gene expression values in the tarsal plates of young and aged mice measured in mRNA microarray experiments.

*Gene* ^1^	Age, 2 ± 1 mo	Age, 32 ± 2 mo	*p*-Value		*Gene* ^1^	Age, 2 ± 1 mo	Age, 32 ± 2 mo	*p*-Value
*Awat1*	16.8	16.1	0.123		*Far1*	15.7	16.1	0.216
*Awat2*	17.9	17.3	0.146		*Far2*	19.2	19	0.72
*Dgat2*	17.1	17.1	no diff.		***Fasn***	**14**	**14.7**	**0.042**
*Dhcr24*	19	19.2	0.8		*Scd1*	19.9	19.9	no diff.
*Dhcr7*	10.3	10	0.803		*Scd2*	17.74	17.93	0.297
***Elovl1***	**17.5**	**15.9**	**0.03**		*Scd3*	18.4	18.9	0.33
*Elovl2*	4.3	4.2	0.72		*Scd4*	18.7	18.8	0.999
***Elovl3***	**17.8**	**16.8**	**0.009**		*Sdr16c5*	14.5	13.6	0.159
*Elovl4*	19.6	19.5	0.499		*Sdr16c6*	17.9	17.3	0.186
*Elovl5*	12	12.5	0.436		*Soat1*	17.7	18	0.424
***Elovl6***	**12.6**	**13.7**	**0.02**		*Soat2*	4.6	5	0.122
***Elovl7***	**15.9**	**15.2**	**0.022**					

^1^ Genes shown in bold were expressed differentially (*p* < 0.05). Main genes of meibogenesis are shown. Additional information on the expression levels of the genes in individual mouse samples can be found in Supplementary Table S5.

## Data Availability

All the data are included in the main manuscript and as Supplementary Materials.

## Supplementary Materials

The supporting information can be downloaded at: https://www.mdpi.com/article/10.3390/ijms241713512/s1.
