# Supplementary material for: Dysregulation of Lipid Metabolism in Aging Meibomian Glands and Its Molecular Markers
Source: Int J Mol Sci. 2023 Aug 31;24(17):13512. doi: 10.3390/ijms241713512 (PMC10488057; doi:10.3390/ijms241713512)
Supplement: Supplementary file 1 [file ijms-24-13512-s001.zip › Suppl Figure 1.pdf]

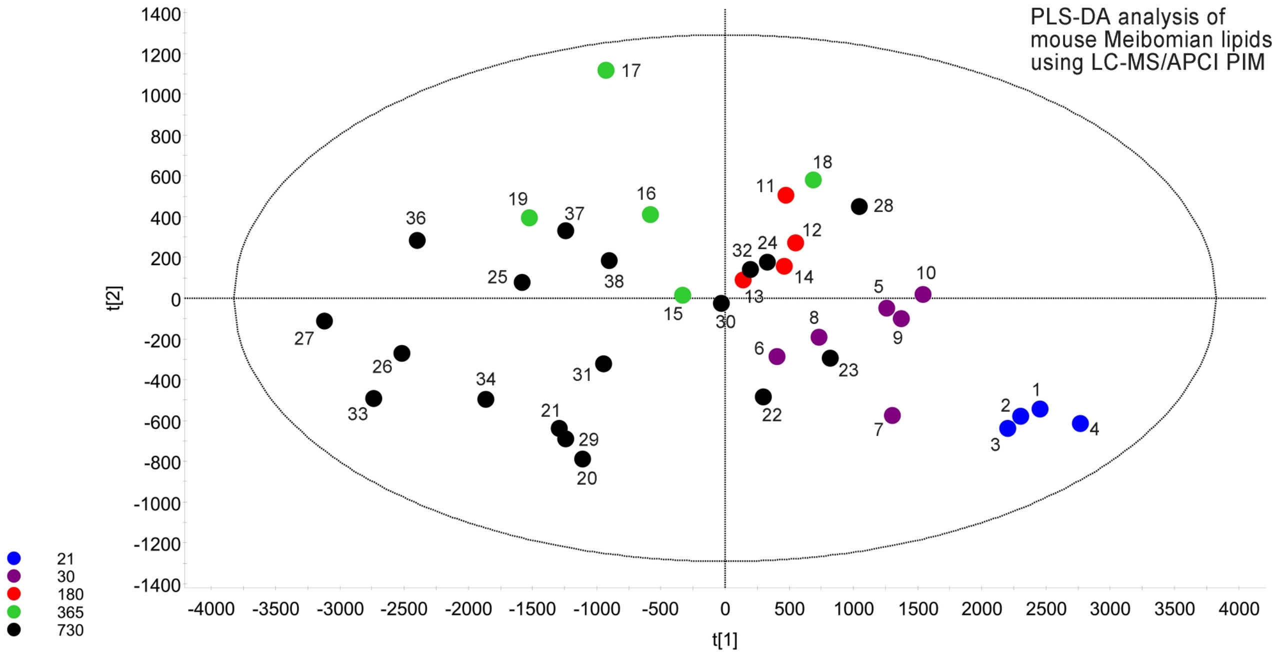

Supplemental Figure S1. A PLS-DA Scores plot of mouse Meibomian lipids analyzed using LC-MS/APCI PIM. The color-coded datapoints are labeled according to Supplemental Table S1. Note a much tighter grouping of the samples originated from younger mice, and a wider spread of samples P365 and P730.
