# Supplementary material for: Dysregulation of Lipid Metabolism in Aging Meibomian Glands and Its Molecular Markers
Source: Int J Mol Sci. 2023 Aug 31;24(17):13512. doi: 10.3390/ijms241713512 (PMC10488057; doi:10.3390/ijms241713512)
Supplement: Supplementary file 1 [file ijms-24-13512-s001.zip › Suppl Table S1.pdf]

Supplemental Table S1. Study animals and sample ID's.

Sample	*Age, days*	*Age group*	*Sex*
1	21	P21	M
2	21	P21	F
3	21	P21	M
4	21	P21	M
5	30	P30	F
6	30	P30	F
7	30	P30	F
8	30	P30	M
9	30	P30	M
10	30	P30	M
11	180	P180	M
12	180	P180	M
13	180	P180	M
14	180	P180	M
15	365	P365	F
16	365	P365	F
17	365	P365	F
18	365	P365	M
19	365	P365	F
20	365	P365	F
39	365	P365 (high cont.)	F
20	730	P730	F
21	730	P730	F
22	730	P730	F
23	730	P730	M
24	730	P730	M
25	730	P730	F
26	730	P730	F
27	730	P730	F
28	730	P730	M
29	730	P730	F
30	730	P730	F
31	730	P730	M
32	730	P730	M
33	730	P730	F
34	730	P730	M
35	730	P730	F
36	730	P730	F
37	730	P730	F
38	730	P730	F
