# Supplementary material for: Dysregulation of Lipid Metabolism in Aging Meibomian Glands and Its Molecular Markers
Source: Int J Mol Sci. 2023 Aug 31;24(17):13512. doi: 10.3390/ijms241713512 (PMC10488057; doi:10.3390/ijms241713512)
Supplement: Supplementary file 1 [file ijms-24-13512-s001.zip › Suppl Table S2.pdf]

Supplemental Table S2. Some of the major Meibomian lipid analytes detected in tarsal plates of mice.

**Protocol**	***m/z* (exp.)**	**Lipid class**	**Molecular formula, detected adduct**
MS PIM	563.5761	WE C38:1	$C_{38}H_{74}O_2$, $(M+H)^+$
	591.6076	WE C40:1	$C_{40}H_{78}O_2$, $(M+H)^+$
	605.62	WE C41:1	$C_{41}H_{80}O_2$, $(M+H)^+$
	619.64	WE C42:1	$C_{42}H_{82}O_2$, $(M+H)^+$
	633.6548	WE C43:1	$C_{43}H_{84}O_2$, $(M+H)^+$
	647.6685	WE C44:1	$C_{44}H_{86}O_2$, $(M+H)^+$
	661.6827	WE C45:1	$C_{45}H_{88}O_2$, $(M+H)^+$
	673.6861	WE C46:2	$C_{46}H_{88}O_2$, $(M+H)^+$
	675.6994	WE C46:1	$C_{46}H_{90}O_2$, $(M+H)^+$
	701.7160	WE C48:2	$C_{48}H_{92}O_2$, $(M+H)^+$
	715.7288	WE C49:2	$C_{49}H_{94}O_2$ $(M+H)^+$
	729.7466	WE C50:2	$C_{50}H_{96}O_2$, $(M+H)^+$
	743.7603	WE C51:2	$C_{51}H_{98}O_2$, $(M+H)^+$
	757.7765	WE C52:2	$C_{52}H_{100}O_2$, $(M+H)^+$
	855.7421	TAG 52:4	$C_{55}H_{98}O_6$, $(M+H)^+$
	857.7573	TAG 52:3	$C_{55}H_{100}O_6$, $(M+H)^+$
	746.7750	WE C50:2	$C_{50}H_{100}NO_2$, $(M+NH_4)^+$
	874.7848	TAG 52:3	$C_{55}H_{104}NO_6$, $(M+NH_4)^+$
	876.8021	TAG 52:2	$C_{55}H_{106}NO_6$, $(M+NH_4)^+$
	1173.1260	Chl-OAHFA	$C_{79}H_{146}NO_4$, $(M+NH_4)^+$
MS NIM	742.5396	PE 36:2	$C_{41}H_{78}NO_{10}P$, $(M-H)^-$
	747.5613	TAG C45:8	$C_{48}H_{77}O_6$, $(M-H)^-$
	835.5319	PI 34:1	$C_{43}H_{80}O_{13}P$, $(M-H)^-$
	863.5624	PI 36:1	$C_{45}H_{84}O_{13}P$, $(M-H)^-$
	311.1685<br>325.1839	Suspected contaminants – alkylbenzene-sulfonates	
