# Supplementary material for: Dysregulation of Lipid Metabolism in Aging Meibomian Glands and Its Molecular Markers
Source: Int J Mol Sci. 2023 Aug 31;24(17):13512. doi: 10.3390/ijms241713512 (PMC10488057; doi:10.3390/ijms241713512)
Supplement: Supplementary file 1 [file ijms-24-13512-s001.zip › Suppl Table S3.pdf]

Supplemental Table S3. Comparison of the P30 and P730 samples signals and signal ratios.

Age group	***Age (days)***	***Sample ID***	***Analyte's m/z***	***673.684***	***701.716***	***715.7288***	***729.7466***	***743.7603***	***757.7765***	***563.5713***	***591.6045***	***605.6126***	***619.6386***	***633.6522***	***647.6685***	***661.6827***	***675.6994***
			LC retention time (min)	***21.46***	***23.87***	***24.58***	***26.29***	***26.99***	***28.83***	***15.23***	***17.24***	***18.28***	***19.35***	***20.48***	***21.6***	***22.8***	***24.04***
				Peak area	***Peak area***	***Peak area***	***Peak area***	***Peak area***	***Peak area***	***Peak area***	***Peak area***	***Peak area***	***Peak area***	***Peak area***	***Peak area***	***Peak area***	***Peak area***
P30	30	5		459013.4	844647	66381.69	422465.8	20080.97	47820.39	31907.53	241706.8	316609.1	1479415	728634.8	954457.5	407041.4	207379.8
P30	30	6		409698.4	773576.4	62467.13	377839.7	21838.74	48606.4	26123.04	198793.5	282773.5	1334397	641534.7	848993.6	362157.6	189321.1
P30	30	7		503080.8	865208.6	60658.64	404356.4	17464.53	42448.93	39698.98	306415.3	364446.5	1564863	708235.8	926159	386944.6	211014.9
P30	30	8		464691	818349.3	62121.8	401227.1	18446.2	43192.54	30026.11	236242.2	308446.8	1403719	652084.2	872029.3	360239.6	189294
P30	30	9		474944.3	885884.7	72589.58	449319.6	23200.34	51916.96	27575.38	220590.5	298576.4	1440127	710248.4	985320.3	417798.5	209020.4
P30	30	10		447918.9	824253	91870.41	417413.5	25488.28	43940.04	25241.98	209469.6	316408.8	1465586	749211.4	977822.6	423118.1	199808.1
P730	730	27		295621.4	450575.1	30646.12	159299.1	7050.367	14977.38	25201.07	152144.9	263764.9	941660.8	355850.4	459094	165048.3	100624.7
P730	730	28		478726.5	780663.3	62860.67	344699.2	19719.77	35252.26	25843.91	187128.2	284359.5	1574486	680956.8	926495.7	365595.5	180819.9
P730	730	29		445043.8	575657.1	33680.48	172628.8	7356.374	13456.09	44009.31	290032.3	367805.5	1379897	505719.5	509551.5	185109.7	125357.2
P730	730	31		457861.8	670722.6	38832.84	217385.5	9123.127	19541.43	36254.73	253332.8	327554.6	1389780	493816.2	541210.4	188901.8	131987.9
P730	730	32		484986.5	763777.1	51064.25	337046.7	14028.51	35182.28	25269.1	203423.9	275759.2	1434660	571170.8	853351	296354.9	173261.2
P730	730	33		377627.3	498859.3	27990.71	153728.2	5927.478	13552.32	30608.68	203006.1	284273.5	1095613	369637.9	425192	141854	96595.8
P730	730	34		405954.8	567437.3	33887.81	182796.5	7662.096	15318.32	31633.29	224777.5	316644.3	1309403	471715.3	513291.1	182395	113968.2
P730	730	35		531078.8	756970.4	54459.4	288257	14791.77	24353.42	31957.77	250223.2	354664.4	1779808	724914.4	858303.8	328819.7	170707.9
P730	730	36		369914.1	540138.6	44445.57	181516.7	9701.314	15667.2	26880.65	175926.1	303427.8	1088791	436406.8	477741.5	175415.5	104055.1
P730	730	37		392168.3	660744.5	47273.73	257463.2	13183.73	25696.08	24462.87	157123.5	255357.6	1235663	511127.8	611683.2	237362.5	141735.5
P730	730	38		391364.1	633007.3	41570.83	211973.4	10795.99	19866.41	29968.74	203427.3	300621.8	1387103	545564	589648.3	231938	143720.5
P730	730	20		505172.1	676046.1	39878.29	198483.2	8702.32	15685.08	32436.8	252985.9	313759	1486991	500146.7	549817.9	180848.8	117234.2
P730	730	21		473652.9	645948.1	40064.78	199671.5	8943.636	15271.23	31846.13	243052.6	333415.9	1436536	509780.7	566855.8	192276.5	122092.8
P730	730	22		453916.1	595511.1	38152.79	200813.6	8897.109	19471.85	41671.25	290313.8	388895.7	1649222	606384.3	646161.2	233702.4	141300.3
P730	730	23		549806.4	779951.8	50222.18	299983.4	12864.95	27325.87	31817.01	251923.3	350913.1	1637222	696180.6	866241.1	322626.1	170429.1
P730	730	24		477294.3	756774	52623.39	329089.4	15008.07	32054.67	25542.34	195629.7	291680.1	1487766	627515.1	866352.6	327114	172172.2
P730	730	25		376118.2	544202.4	34500.71	187579.3	8467.631	16066.11	30563.14	189658.7	279561.7	1304268	499717.8	548501.9	207554.5	125563.8
P730	730	26		368940.3	486102.8	29925.43	143632.9	7008.175	11837.41	31648.09	199454.1	294067.3	1163888	435499	437323.1	162320.5	97574.02

Analyte's m/z	***673.684***	***701.716***	***715.7288***	***729.7466***	***743.7603***	***757.7765***	***563.5713***	***591.6045***	***605.6126***	***619.6386***	***633.6522***	***647.6685***	***661.6827***	***675.6994***
RT, min	***21.46***	***23.87***	***24.58***	***26.29***	***26.99***	***28.83***	***15.23***	***17.24***	***18.28***	***19.35***	***20.48***	***21.6***	***22.8***	***24.04***
Sample group	***Average peak area in the group***	***Average peak area in the group***	***Average peak area in the group***	***Average peak area in the group***	***Average peak area in the group***	***Average peak area in the group***	***Average peak area in the group***	***Average peak area in the group***	***Average peak area in the group***	***Average peak area in the group***	***Average peak area in the group***	***Average peak area in the group***	***Average peak area in the group***	***Average peak area in the group***
**P30**	459891.1333	835319.8333	69348.20833	412103.6833	21086.51	46320.87667	30095.50333	235536.3167	314543.5167	1448017.833	698324.8833	927463.7167	392883.3	200973.05
**P730**	435291.5389	632393.8278	41782.22111	225891.5333	10512.91206	20587.52278	30978.60444	217975.7722	310362.55	1376819.878	530116.8944	624823.1167	229179.8722	134955.5733
**Ratio P730/P30**	0.946509961	0.757067895	0.602498927	0.548142476	0.498561026	0.444454515	1.029343291	0.92544443	0.986707828	0.950830747	0.759126457	0.673690092	0.583328108	0.671510799
