# Supplementary material for: Dysregulation of Lipid Metabolism in Aging Meibomian Glands and Its Molecular Markers
Source: Int J Mol Sci. 2023 Aug 31;24(17):13512. doi: 10.3390/ijms241713512 (PMC10488057; doi:10.3390/ijms241713512)
Supplement: Supplementary file 1 [file ijms-24-13512-s001.zip › Suppl Table S4.pdf]

Supplemental Table S4. Proposed markers of aging and their signal ratios

Sample ID	Age group	Age (days)	m/z 647.6685 Signal	m/z 855.7421 Signal	Ratio 647/855	Ratio, Mean	Ratio, SD
**1**	P21	21	1009490	32193.29	31.35715548	**30.96167471**	**6.385389513**
**2**	P21	21	938182	24962.76	37.58326403		
**3**	P21	21	960969.4	43147.88	22.27153223		
**4**	P21	21	1044837	32016.09	32.63474709		
**5**	P30	30	930138.9	42828.69	21.71765935	**17.38976672**	**5.285803496**
**6**	P30	30	803634.8	75913.37	10.58620899		
**7**	P30	30	901097.4	41286.69	21.82537278		
**8**	P30	30	857961.3	78603.14	10.91510212		
**9**	P30	30	975731.6	52900.75	18.44457026		
**10**	P30	30	998731.9	47901.53	20.84968685		
**11**	P180	180	926828.6	119053	7.785008358	**7.249372553**	**0.654281416**
**12**	P180	180	876931.7	119383.4	7.345507834		
**13**	P180	180	759749.1	120488.4	6.305578794		
**14**	P180	180	820074.9	108455.5	7.561395227		
**15**	P365	365	654368.9	95400.35	6.859187624	**6.010178014**	**2.035276989**
**19**	P365	365	588130.2	163124.2	3.605413544		
**16**	P365	365	742018.9	129235.1	5.741620504		
**17**	P365	365	864840.1	177164.7	4.881559927		
**18**	P365	365	947246.4	105682.8	8.963108472		
**27**	P730	730	503762.2	392493.2	1.28349281	**4.929945565**	**3.797141849**
**29**	P730	730	479655.7	134047.8	3.578243731		
**36**	P730	730	532302	269452.2	1.975496953		
**37**	P730	730	651119.7	244986.5	2.657777878		
**38**	P730	730	627054.3	175990.6	3.562998819		
**25**	P730	730	573688.4	208284.5	2.75434994		
**26**	P730	730	441278.5	246026.6	1.793621096		
**28**	P730	730	983465.4	88726.35	11.084254		
**31**	P730	730	535730.7	141272.4	3.792182337		
**32**	P730	730	847212	124732.2	6.792247711		
**33**	P730	730	389350.8	268557.4	1.449786154		
**34**	P730	730	459908.2	201630.8	2.280942197		
**20**	P730	730	509538	173237.5	2.94126849		
**21**	P730	730	536028.5	192127.8	2.789958038		
**22**	P730	730	678902.8	51986.44	13.05922852		
**23**	P730	730	854444.5	71153.7	12.00843386		
**24**	P730	730	866352.6	122238.7	7.087383946		
**30**	P730	730	725171.6	92409.7	7.847353687		

Sample ID	Age group	Age (days)	m/z 742.5397 Signal	m/z 747.5614 Signal	Ratio 742/747	Ratio, Mean	Ratio, SD
5	P30	30	1885591	813173.3	2.318805844	**2.463235134**	**0.289857975**
6	P30	30	1615309	822947.4	1.962833834		
7	P30	30	1804385	660391.3	2.732296746		
8	P30	30	1838876	750337.5	2.450731837		
9	P30	30	2034988	771948	2.636172385		
10	P30	30	1805982	674233.6	2.678570157		
11	P180	180	1648233	581358.1	2.835142402	**2.146498213**	**0.499011013**
12	P180	180	1433042	670157.9	2.138364705		
13	P180	180	1446870	870177.1	1.662730495		
14	P180	180	1809818	928228.3	1.949755249		

39 (high contam. in PIM)	P365	365	1002334	827770.5	1.210883935	**1.595113011**	**0.825558605**
15	P365	365	878080	1009307	0.869983068		
16	P365	365	1183491	680910.5	1.738100675		
17	P365	365	1885998	771662.5	2.44407108		
18	P365	365	2098733	747130.4	2.809058499		
19	P365	365	878300.8	1021900	0.859478227		
27	P730	730	962911.8	780181.2	1.23421559	**1.190016691**	**0.516889076**
31	P730	730	964613.4	1284545	0.750937803		
32	P730	730	1515614	991869.6	1.528037557		
33	P730	730	668725.4	1025499	0.652097564		
34	P730	730	836868.7	1040059	0.804635795		
20	P730	730	728751.3	784483.1	0.928957297		
21	P730	730	1047911	1035271	1.012209364		
22	P730	730	962416	1541443	0.624360421		
23	P730	730	1740889	860832.9	2.022330931		
24	P730	730	1753060	859931.1	2.038605186		
25	P730	730	1647439	1102854	1.493796096		
