# Supplementary material for: Dysregulation of Lipid Metabolism in Aging Meibomian Glands and Its Molecular Markers
Source: Int J Mol Sci. 2023 Aug 31;24(17):13512. doi: 10.3390/ijms241713512 (PMC10488057; doi:10.3390/ijms241713512)
Supplement: Supplementary file 1 [file ijms-24-13512-s001.zip › Suppl Table S5.pdf]

Supplemental Table S5. Transcriptomic analysis of the genes of meibogenesis in tarsal plates of young and aged mice.

Transcript ID	O, Avg (log2)	Y, Avg (log2)	O, Standard Deviation	Y, Standard Deviation	Fold Change	P-val	FDR P-val	Gene Symbol	Description	Chromosome	Strand
TC0X00000954.mm.1	16.11354	16.75056	0.1732209	0.6393266	-1.555109	0.1232317	0.4032081	**Awat1**	acyl-CoA wax alcohol acyltransferase 1	chrX	+
TC0X00002703.mm.1	17.25481	17.93608	0.02991082	0.7648185	-1.603551	0.1460657	0.4231403	**Awat2**	acyl-CoA wax alcohol acyltransferase 2	chrX	-
TC1500001777.mm.1	7.938121	7.580911	0.1427068	0.6101214	1.280946	0.1745992	0.4454364	**Dgat1**	diacylglycerol O-acyltransferase 1	chr15	-
TC0700003793.mm.1	17.05849	17.04835	0.1357732	0.2874537	1.007053	0.8141475	0.8895981	**Dgat2**	diacylglycerol O-acyltransferase 2	chr7	-
TC0X00000953.mm.1	5.289975	5.180963	0.2208965	0.6492085	1.07849	0.5298291	0.6996225	**Dgat2l6**	diacylglycerol O-acyltransferase 2-like 6	chrX	+
TC0700002102.mm.1	9.963264	10.29541	0.1335767	0.6053552	-1.258882	0.8029385	0.8826084	**Dhcr7**	7-dehydrocholesterol reductase	chr7	+
TC0400001107.mm.1	19.18252	18.9657	0.02655839	0.4505676	1.162171	0.7995633	0.8805361	**Dhcr24**	24-dehydrocholesterol reductase	chr4	+
TC0400001307.mm.1	15.89529	17.50234	0.03415795	1.016597	-3.046284	0.02995662	0.3171264	**Elovl1**	elongation of very long chain fatty acids (FEN1/Elo2, SUR4/Elo3, yeast)-like 1	chr4	+
TC1300001859.mm.1	4.190957	4.295771	0.1853108	0.5227287	-1.075356	0.7178754	0.8279817	**Elovl2**	elongation of very long chain fatty acids (FEN1/Elo2, SUR4/Elo3, yeast)-like 2	chr13	-
TC1900000684.mm.1	16.76901	17.81568	0.2030525	0.5320604	-2.065757	0.008665898	0.2752776	**Elovl3**	elongation of very long chain fatty acids (FEN1/Elo2, SUR4/Elo3, yeast)-like 3	chr19	+
TC0900002767.mm.1	19.45849	19.61726	0	0.2124378	-1.116332	0.4993049	0.6781186	**Elovl4**	elongation of very long chain fatty acids (FEN1/Elo2, SUR4/Elo3, yeast)-like 4	chr9	-
TC0900001042.mm.1	12.50803	12.04144	0.1182687	1.095475	1.38184	0.4361134	0.6350995	**Elovl5**	ELOVL family member 5, elongation of long chain fatty acids (yeast)	chr9	+
TC0300001306.mm.1	13.66852	12.54919	0.3364016	0.7112905	2.17246	0.01958207	0.3039622	**Elovl6**	ELOVL family member 6, elongation of long chain fatty acids (yeast)	chr3	+
TC1300001204.mm.1	15.18644	15.90316	0.09961495	0.3928966	-1.643431	0.02159803	0.306678	**Elovl7**	ELOVL family member 7, elongation of long chain fatty acids (yeast)	chr13	+
TC0700001666.mm.1	16.12214	15.66297	0.2343304	0.4276258	1.374751	0.2161895	0.4756214	**Far1**	fatty acyl CoA reductase 1	chr7	+
TC0600001751.mm.1	18.96681	19.20119	0.02529769	0.6623121	-1.176402	0.7199287	0.8294371	**Far2**	fatty acyl CoA reductase 2	chr6	+
TC1100004233.mm.1	14.69113	13.99664	0.04108461	0.6846483	1.618308	0.04241927	0.3300862	**Fasn**	fatty acid synthase	chr11	-
TC1900001562.mm.1	19.90293	19.91072	0	0.6975101	-1.005413	0.4892145	0.6718435	**Scd1**	stearoyl-Coenzyme A desaturase 1	chr19	-
TC1900000646.mm.1	17.93418	17.74274	0.03740957	0.3133449	1.1419	0.2970971	0.5345872	**Scd2**	stearoyl-Coenzyme A desaturase 2; microRNA 5114	chr19	+
TC1900000645.mm.1	18.85817	18.44214	0.01358865	1.282955	1.334257	0.3297917	0.5590865	**Scd3**	stearoyl-coenzyme A desaturase 3	chr19	+
TC1900000647.mm.1	18.76632	18.7017	0.09532184	0.5634965	1.045812	0.9987685	0.9991775	**Scd4**	stearoyl-coenzyme A desaturase 4	chr19	+
TC0400002136.mm.1	13.62367	14.4769	0.2492366	0.7462798	-1.806532	0.1587546	0.4326673	**Sdr16c5**	short chain dehydrogenase/reductase family 16C, member 5	chr4	-
TC0400002137.mm.1	17.25538	17.94581	0.1764968	0.6171432	-1.613764	0.1855069	0.4540502	**Sdr16c6**	short chain dehydrogenase/reductase family 16C, member 6	chr4	-
TC0100003319.mm.1	17.98055	17.66525	0.0346699	0.5156729	1.244274	0.4242113	0.6262359	**Soat1**	sterol O-acyltransferase 1	chr1	-
TC1500001090.mm.1	4.95733	4.566874	0.06579777	0.6412525	1.310807	0.1224948	0.4026941	**Soat2**	sterol O-acyltransferase 2	chr15	+

NOTES. Expression Analysis Settings:
• Gene-Level Fold Change < -2 or > 2
• Gene-Level P-Value < 0.05
• Anova Method: ebayes

Array Type: MTA-1_0
Analysis Type: Expression (Gene)
Analysis Version: version 2
Pos vs Neg AUC Threshold: 0.7
Genome Version: mm10 (Mus musculus)
Annotation: MTA-1_0.r3.na36.mm10.a1.transcript.csv
Map File: MTA-1_0_MappingFile.r1.map
Condition (Comparison): O; Y

Study animals: Wild type C57/Bl6 mice;
Mouse age = 32+/-2 mo (O);
Mouse age = 2+/1 mo (Y);

Elovl4 and Scd1 are among the most highly expressed genes in mouse tarsal plates. The analysis produced three identical expression levels for each of these genes in three samples O-1, O-2, and O-3 obtained from aged mice, which resulted in p-values of 0 for both genes.

			Mouse Y-1 (male)	Mouse Y-2 (female)	Mouse Y-3 (female)	Mouse Y-4 (male)	Mouse Y-5 (male)	Mouse O-1 (male)	Mouse O-2 (male)	Mouse O-3 (male)
**Group**	**Start**	**Stop**	**Y-1.sst-rma-gene-full.chp Signal**	**Y-2.sst-rma-gene-full.chp Signal**	**Y-3.sst-rma-gene-full.chp Signal**	**Y-4.sst-rma-gene-full.chp Signal**	**Y-5.sst-rma-gene-full.chp Signal**	**O-1.sst-rma-gene-full.chp Signal**	**O-2.sst-rma-gene-full.chp Signal**	**O-3.sst-rma-gene-full.chp Signal**
Coding	100572247	100578206	16.88715	16.47738	16.53892	17.094	17.59972	16.29169	16.10802	15.94546
Coding	100402221	100442717	18.80317	17.32479	17.65504	18.32366	18.43635	17.25679	17.28263	17.22298
Multiple_Complex	76502015	76511951	6.346151	7.570967	7.279724	7.747017	8.167194	8.071044	7.94742	7.786446
Multiple_Complex	99153658	99182713	17.19548	16.72398	16.95423	17.24436	17.41164	17.05735	17.05973	16.82338
Multiple_Complex	100524838	100546144	3.716151	4.985201	5.423893	5.328321	5.361608	5.295478	5.284901	4.907695
Multiple_Complex	143823145	143848410	8.989134	9.913491	10.50606	10.08272	10.44176	10.15471	9.899292	9.959178
Coding	106561038	106589113	19.93151	18.6071	18.89428	19.06241	19.19288	19.20428	19.15188	19.18563
Multiple_Complex	118428093	118432953	17.95915	17.19588	17.49849	17.42643	17.59242	15.91364	15.89736	15.84804
Coding	41182382	41220405	3.058044	4.141683	4.194306	4.432929	4.447953	4.419455	4.168806	4.057695
Coding	46131897	46135694	18.42064	17.56257	17.61362	17.93872	18.30398	16.74973	16.993	16.58976
Coding	83778692	83806305	19.93151	19.50347	19.52449	19.70613	19.74662	19.45849	19.45849	19.45849
Multiple_Complex	77917364	77984519	10.57613	13.36317	13.31473	11.92176	11.91287	12.63389	12.39806	12.50019
Coding	129532386	129638495	12.4735	11.48927	12.5481	12.2905	13.01457	13.94062	13.27765	13.70842
Coding	108214404	108287109	15.80723	15.64626	15.97771	16.18785	16.50585	15.19808	15.26801	15.07149
Multiple_Complex	113513834	113571511	15.41853	15.71083	15.52735	16.07934	16.39729	15.89297	16.36146	16.11646
Multiple_Complex	148047259	148182760	19.93151	19.14873	19.14873	19.24483	19.26634	18.99835	18.96385	18.94905
Multiple_Complex	120805940	120824547	14.00727	12.91757	13.05076	14.11415	14.60961	14.71577	14.63647	14.69473
Multiple_Complex	44394450	44407709	19.93151	19.90829	19.90829	19.91314	19.91314	19.90293	19.90293	19.90293
Multiple_Complex	44293676	44306864	17.80788	17.7694	17.88337	17.64634	17.65293	17.93436	17.86939	17.934
Coding	44203288	44244016	19.73074	18.21796	18.6421	18.32887	18.57189	18.85718	18.85925	18.83475
Multiple_Complex	44333092	44346743	19.67463	18.56731	18.75018	18.6672	18.81523	18.80557	18.62415	18.7656
Multiple_Complex	3995936	4019663	15.03942	14.06698	14.3245	14.60479	15.1261	13.64757	14.05219	13.59775
Coding	4055929	4077522	18.56471	17.52654	17.77942	18.14112	18.28517	17.27003	17.55931	17.23948
Multiple_Complex	156428108	156474328	17.90225	17.20863	17.31813	18.13107	18.42408	17.93236	17.9989	17.98252
Coding	102150526	102163474	3.179831	5.04288	4.781711	4.471116	4.393638	4.964327	5.064648	4.940732
